# *Runx1* overexpression induces early onset of intervertebral disc degeneration

**DOI:** 10.18632/aging.206316

**Published:** 2025-09-08

**Authors:** Takanori Fukunaga, Martha Elena Diaz-Hernandez, John G. Heller, Changli Zhang, Hicham Drissi

**Affiliations:** 1 Musculoskeletal Institute, Emory University School of Medicine, Atlanta, GA 30329, USA; 2 Atlanta VA Medical Center, Decatur, GA 30033, USA

**Keywords:** cell senescence, aging, Runx1, nucleus pulposus, intervertebral disc degeneration

## Abstract

Intervertebral disc degeneration (IDD) is closely associated with aging. Although the Runt-related transcription factor 1 (*RUNX1*) is well known for its role in skeletal development and other musculoskeletal related diseases such as osteoarthritis, its involvement in IDD pathogenesis remains elusive. In this study, we examined the function of *Runx1* specifically in the nucleus pulposus (NP) *in vivo*. To achieve NP-specific postnatal overexpression of *Runx1*, we crossed *Krt19*^CreERT^ mice with *Rosa26*-*Runx1* transgenic mice previously generated in our laboratory. Mice with NP specific *Runx1* overexpression displayed early onset and progressive disc degeneration beginning at 5 months of age. This was characterized by a phenotypic shift from notochordal cells to hypertrophic chondrocyte-like cells, accompanied by extracellular matrix remodeling, including reduced expression of aggrecan and type II collagen as well as increased type X collagen. In addition, NP cells from these transgenic mice showed increased expression of senescence markers P16 and P21 without significant changes in apoptosis levels. Notably, the severity of degeneration correlated with the number of tamoxifen injections, suggesting a direct association between the level of *Runx1* expression and IVD degeneration. Early histological signs of degeneration in *Runx1* overexpression mice highlighted its potential role as a key IDD initiator. Taken together, these findings reveal a novel role of Runx1 in maintaining disc health and regulating age-related degenerative processes.

## INTRODUCTION

The intervertebral disc (IVD) is a complex structure that consists of the nucleus pulposus (NP), derived from the notochord, which is surrounded by the annulus fibrosus (AF) and interconnected by two cartilage endplates (CEP) [1]. Resident NP cells synthesize extracellular matrix (ECM), mostly comprised of type II collagen and proteoglycans, which are essential for maintaining the mechanical properties of the IVD [2, 3]. Since the activity of NP cells is central to disc function and their repair capacity declines with degeneration, NP cells are considered to play a pivotal role in maintaining the physiology of the IVD [4].

Intervertebral disc degeneration (IDD) is characterized by increased degradation of existing NP matrix due to the elevated expression of matrix metalloproteinases and inflammatory factors. Furthermore, IDD disrupts matrix production [5], with one of the earliest significant changes being the gradual disappearance of NP cells and their differentiation into chondrocyte-like cells (CLCs) [6, 7]. While healthy NP tissue typically exhibits uniform extracellular staining for type II collagen, CLCs in IDD express pericellular immunostaining of type II collagen [7, 8]. The replacement of NP cells with CLCs results in an imbalance between anabolism and catabolism within the IVD microenvironment [9], leading to degradation of ECM proteins and reduced secretion of type II collagen by NP cells [10].

IDD can begin early in life and has been shown to be initiated during childhood. In a previous study, 35% of subjects aged 13–20 years demonstrated IDD on MRI, as detected by signal intensity changes on T2-weighted images [11]. However, the prevalence of IDD increases significantly with age, with a notable 50% difference in occurrence between individuals in their 50s and those in their 70s [12]. Beyond causing immediate discomfort, the progression of IDD is associated with various spinal disorders including herniation and scoliosis. In naturally aging mice, mild degenerative changes were seen as early as 12 months (M) of age, with the severity of disc degeneration markedly increased at 24M and 36M [13]. However, while IDD is recognized as an age-related process of the intervertebral disc, its pathogenesis is complex and influenced by various factors, including inflammation, oxidative stress, mechanical stress, and genetic factors [14–16]. Nevertheless, despite these known considerations, the mechanisms underlying the onset and development of these changes during aging remain elusive.

The process of disc aging can be categorized into three phases [4]. Aging first triggers biomolecular damage resulting from exposure to metabolic stress. These biomolecular changes subsequently cause aberrant cellular responses that, when dysregulated, exacerbate disc tissue damage. Eventually, the accumulated damage leads to structural changes [4]. Among these aberrant cellular responses, senescence of NP cells is reported to increase in aging as well as in degenerating IVDs and is considered a major cause of IDD [17, 18]. The early step of cell senescence in IDD is typically characterized by cells exiting their cell cycle and entering a stable cell-cycle arrest phase through sustained activation of the p16 and/or p53–p21 pathways [19]. Senescent NP cells subsequently secrete inflammatory mediators such as nuclear factor kappa-light-chain-enhancer of activated B cells (NF-κB), along with matrix metallopeptidase 9 (MMP9) and matrix metallopeptidase 13 (MMP13). These factors disrupt the metabolic balance of the ECM and accelerate IDD [20–24]. Additionally, the NF-κB pathway was shown to promote NP cell senescence [25].

The Runt-related transcription factor 1 (RUNX1) was initially identified as acute myeloid leukemia factor 1 (AML1) in patients with acute myeloid leukemia [26]. Since its discovery, RUNX1 has been recognized for its role in regulating development, maintaining homeostasis, and contributing to pathogenesis [27–29]. Its pleiotropic functions have been progressively revealed beyond the hematopoietic and into mesenchymal and other tissues [30–33]. In musculoskeletal tissues, *Runx1* is expressed in pre-chondrogenic mesenchyme at embryonic stages, where it plays a role in the early events of endochondral and intramembranous bone formation [34]. Sato et al*.* induced *Runx1* expression in chondrocytes using the *Col2a1* promoter, resulting in mice that developed scoliosis and IVD degeneration with age, characterized by extracellular matrix remodeling and hypertrophic chondrocytes in the IVD [35]. However, this overexpression was not specific to IVD and occurred prenatally; therefore, its effects on early spine development likely contributed to IVD degeneration. Additionally, our prior network analyses, based on human IVD microarray datasets, identified *RUNX1* as a potential regulator in IDD [36], indicating the need for a more targeted exploration of its role in IDD.

In this study, we found that the postnatal NP-specific overexpression of *Runx1* induced early onset of age-related IVD degeneration in a dose-dependent manner, providing a novel insight into the pathological role of *Runx1* in disc health. Our findings indicated that sustained expression of *Runx1* led to a shift of NP cells into a mature chondrocyte phenotype, disruption of ECM integrity, and premature cellular senescence. Together, our findings suggest a role for *Runx1* in advancing disc degeneration through acceleration of disc cell aging. Thus, we postulate that *Runx1* may be a therapeutic target to slow down aging-induced disc degeneration.

## RESULTS

### Nucleus pulposus specific overexpression of *Runx1* in mice

**Figure 1:**
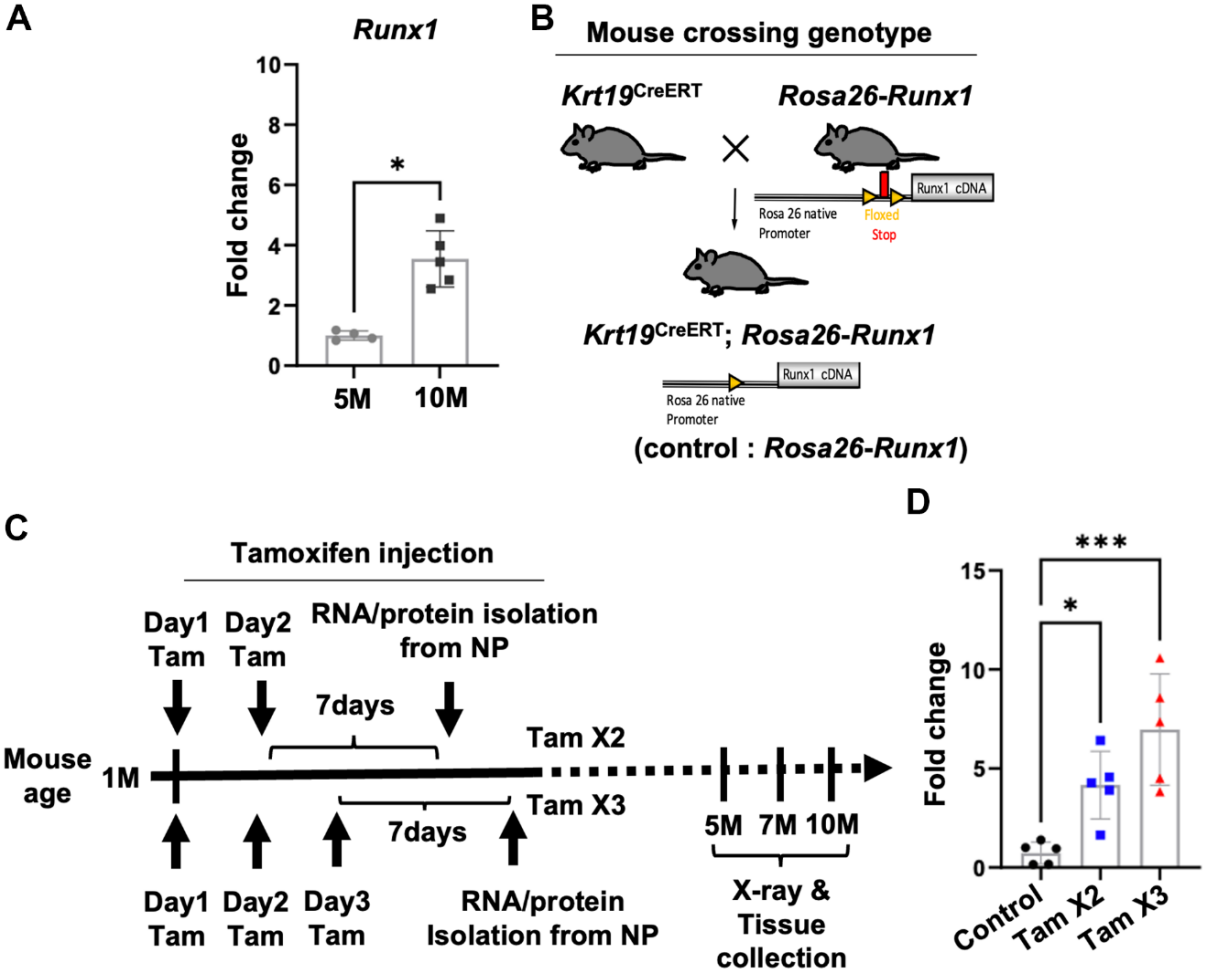
**Conditional upregulation of Runx1 expression in Krt19CreERT; Rosa26-Runx1 mice following Tamoxifen Injections.** (**A**) The gene expression of Runx1 was increased in the NP of control mice at 10M compared to 5M. n = 4~5 mice/group. Student’s t-test was performed. * p < 0.05. (**B**) Generation of double transgenic Krt19CreERT; Rosa26-Runx1 mice. Upon tamoxifen injection, Runx1 was specifically overexpressed in the NP tissues. (**C**) Tamoxifen was administered intraperitoneally at 4 weeks of age. In the Tam X2 group, tamoxifen was injected once a day for two consecutive days while in the Tam X3 group, it was injected once a day for three consecutive days. Runx1 overexpression was verified by Q-PCR using RNAs isolated from NP tissues collected seven days after the final injection in each group. For X-ray and histological analyses, mice were sacrificed at 5, 7, and 10 months (M) of age. (**D**) Quantitative PCR analysis revealed a significant upregulation in the gene expression of Runx1 in Tam X2 and Tam X3 groups compared to the control. n = 5 mice/group. One-way ANOVA followed by Tukey’s post-test was performed. (*** p < 0.001, * p < 0.05). Tam X2 refers to Krt19CreERT; Rosa26-Runx1 mice injected with tamoxifen for two consecutive days and Tam X3 refers to Krt19CreERT; Rosa26-Runx1 injected with tamoxifen for three consecutive days.

Our previous transcriptomic analyses indicated that *RUNX1* was a putative regulator in the pathogenesis of IVD degeneration [36]. In this study, we further observed a significant upregulation of *Runx1* at 10M compared to 5M (Figure 1A), suggesting its involvement in IVD aging/degeneration over time. To explore the function of *Runx1* in IVD degeneration, we generated NP specific *Runx1* overexpression mice by crossing the Tamoxifen inducible *Krt19*^CreERT^ mice with our *Rosa26*-*Runx1* transgenic mice [30], which we named *Krt19*^CreERT^; *Rosa26*-*Runx1* mice (Figure 1B). The *Krt19*^CreERT^; *Rosa26*-*Runx1* mice without tamoxifen induction did not display any overt changes in their axial skeletal phenotype compared to the control mice. We induced *Runx1* overexpression at two different levels by performing two (Tam x2) and three (Tam x3) tamoxifen IP injections in these mice at 4-weeks of age and verified *Runx1* overexpression in NP tissues seven days after final tamoxifen injections (Figure 1C). The qPCR analyses revealed a significant increase in the relative expression level of *Runx1* in the NPs isolated from *Krt19*^CreERT^; *Rosa26*-*Runx1* mice, with a 4.2-fold and 7.0-fold elevation in Tam X2 and Tam X3 groups, respectively, compared to NPs from control mice (Figure 1D). Consistent with the changes in gene expression, the protein expression levels of RUNX1 were also dose-dependently increased with the times of tamoxifen injections (Supplementary Figure 1; Fold change: Tam X2 = 1.79, Tam X3 = 2.49). These data demonstrate that the expression of *Runx1* was increased dose-dependently in the NP compartment *in vivo*.

### Decreased disc height index (DHI) in *Runx1* overexpression mice

**Figure 2:**
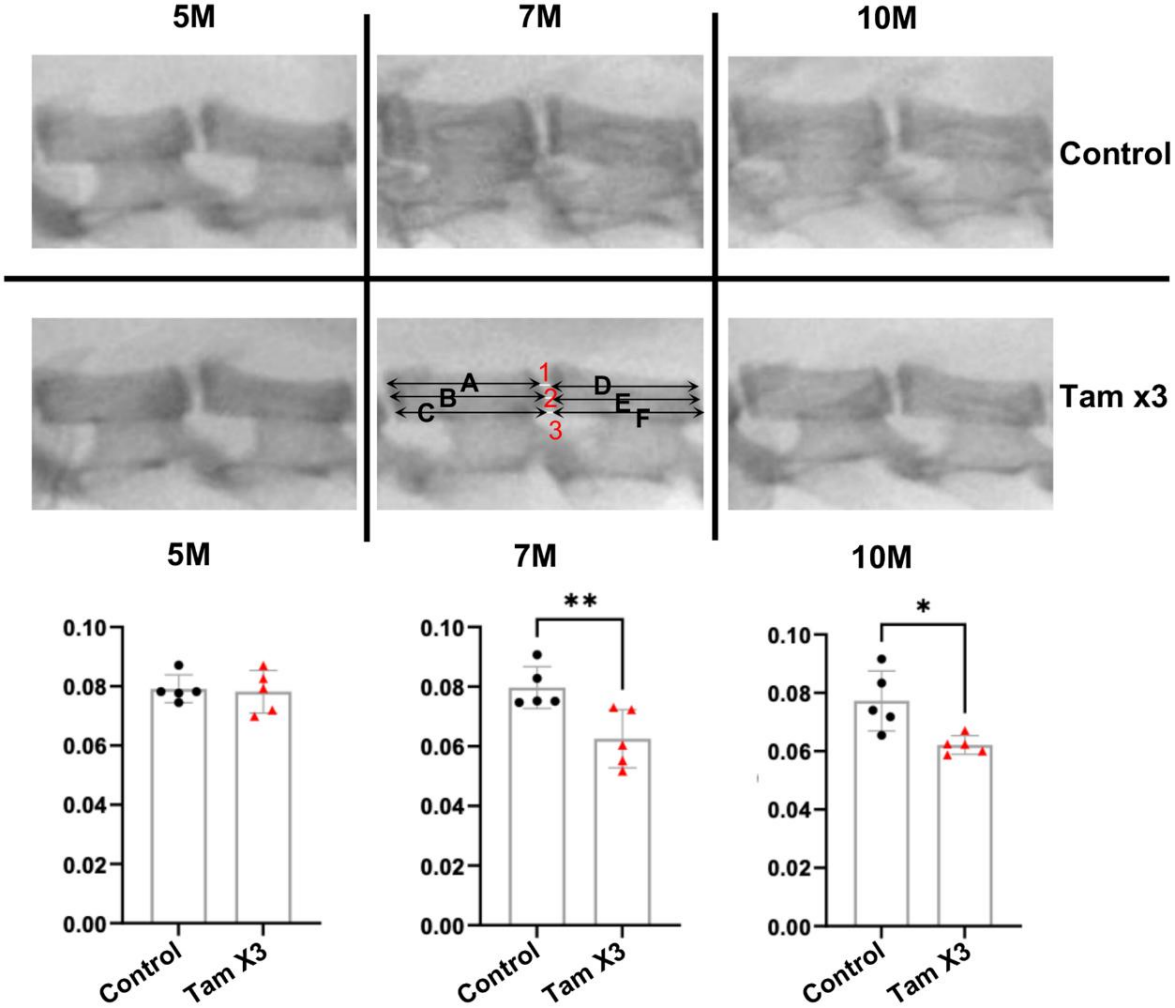
**Runx1 overexpression reduced disc height index at 7M and 10M.** Disc height index (DHI) was determined according to the following equation: DHI = 2 x (DH1 + DH2 + DH3) / (A + B + C + D + E + F). DH represents the disc height between adjacent vertebrae and the letter represents the length of vertebral bone. The DHI at the L4/5 level showed no significant alteration at 5M. However, a substantial reduction was evident in the Tam X3 group at both 7M and 10M, compared to the control group. n = 5 mice /group. Student t-test was performed. (** p < 0.01, * p < 0.05).

A reduced DHI is recognized as an indicator of IDD [37]. To investigate the degenerative effects of *Runx1* overexpression on the intervertebral disc, we measured DHI in the lumbar spine L4-5 at 5M, 7M, and 10M (Figure 2). At 5M, DHI was not significantly changed between the two genotypes; however, at 7M (Figure 2; Fold change: 0.78) and 10M (Figure 2; Fold change: 0.8), it was remarkably decreased in *Runx1* overexpressing mice compared to control mice. These findings suggest that overexpression of *Runx1* in the NP may accelerate IDD over time.

### *Runx1* overexpression enhanced age-related IDD

**Figure 3:**
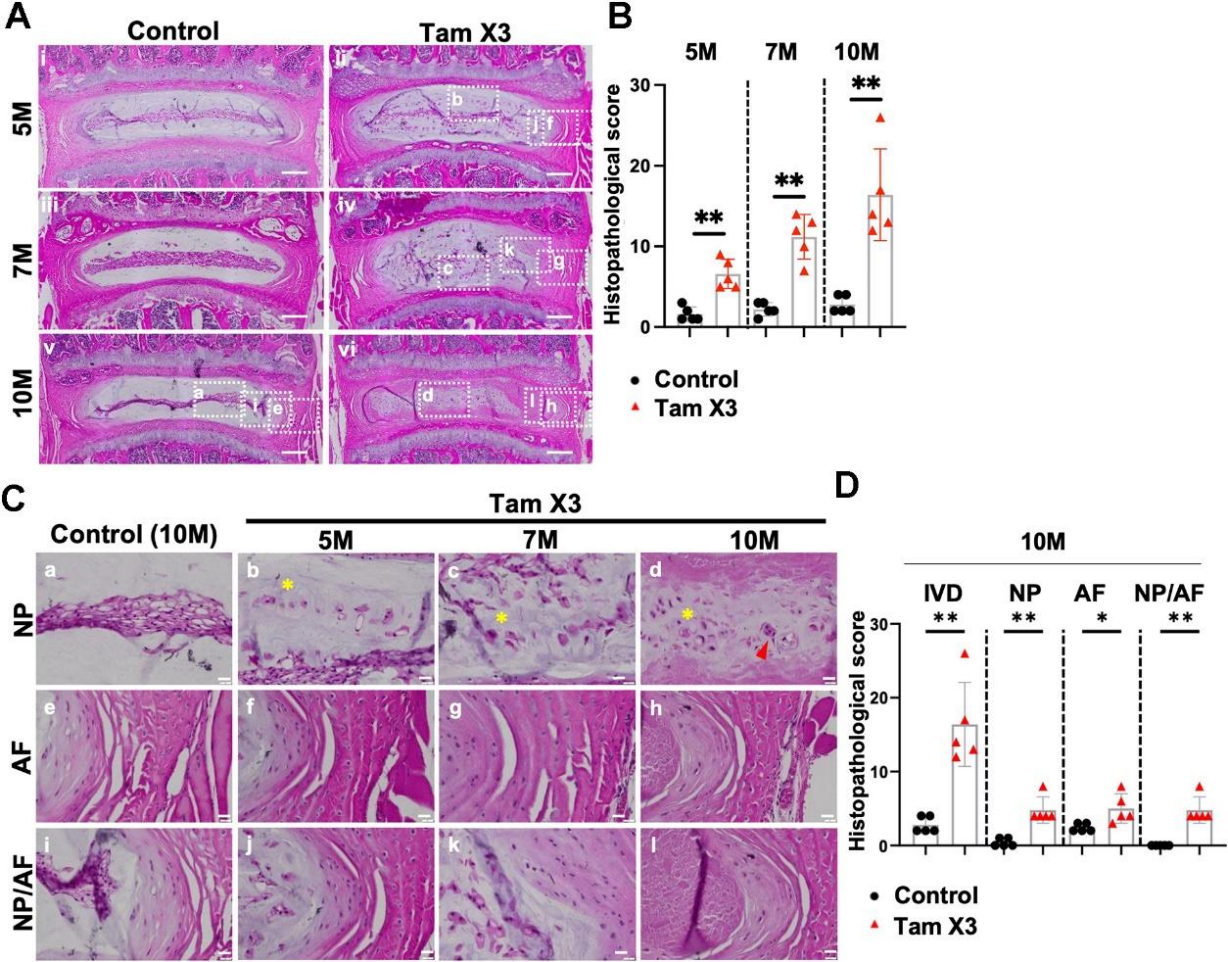
**Runx1 overexpression induced age-related intervertebral disc degeneration.** (**A**) Representative images of Hematoxylin and eosin (H&E) staining in the IVDs of control and Runx1 overexpression (Tam X3) mice at 5M, 7M, and 10M. Control mice showed a highly organized IVD structure. The IVDs of Runx1 overexpression (Tam X3) mice showed progressive IVD structure degeneration over time. Scale bar = 200 μm. (**B**) Total IVD histopathological scores in the IVDs of control and Runx1 overexpression (Tam X3) mice at 5M, 7M, and 10M. Student’s t-test was performed. (** p < 0.01). To compare the effects of different Runx1 expression levels induced by two (Tam X2) or three times (Tam X3) of Tamoxifen injections, we also scored the histological sections of the Tam X2 group and compared them with control and Tam X3 images. Note that the data presented in Supplementary Figure 2 used the same representative images for control and Tam X3 groups as in Figure 3. Since our primary objective was to assess the consequence of Runx1 overexpression on disc aging, we elected to focus the rest of our studies on the highest Runx1 overexpressing mice, which corresponds to the Tam X3 group. (**C**) Higher magnification of H&E images in the NP, AF and NP/AF boundary in the control and Runx1 overexpression (Tam X3) mice. The control NP comprised of tightly connected notochordal cells in the center. The AF tissue was well organized with a distinct boundary between NP and AF tissues. The IVDs of Runx1 overexpression mice showed multiple nuclei in NP cells and the appearance of cell clusters (d) and disorganized extracellular matrix in the NP, with the presence of round-shaped cells in the inner AF over time. Scale bar = 20 μm. Asterisk indicates disorganized extracellular matrix. Arrowhead indicates cell clusters. (**D**) Compartment-specific histopathological scores in the IVDs of control and Runx1 overexpression (Tam X3) mice at 10M. The scores were significantly increased in all compartments of Runx1 overexpression mice at 10M, compared to the control. n = 5 mice/group. Student’s t-test was performed (** p < 0.01, * p < 0.05).

To evaluate the effects of *Runx1* overexpression on disc health, we examined the morphological and structural changes in the IVD of control and *Runx1*-overexpressing mice at 5M, 7M, and 10M. Histopathological analyses of H&E staining demonstrated a well-organized IVD structure in the control group across all timepoints. In contrast, Tam X2 and Tam X3 groups exhibited progressive IVD degeneration in a dose-dependent manner, with Tam X3 group showing more severe IVD degenerative changes at 10M (Supplementary Figure 2). These changes were reflected by higher degeneration scores in the Tam X3 group at 10M (Figure 3A, 3B; Fold change: 5M = 4.13, 7M= 5.09, 10M= 5.86). To gain further insight into the specific tissue alterations in the Tam X3 group, we performed histopathological analyses of NP, AF and NP/AF boundary regions. In the control group, NP cells displayed a reticular and evenly spread cell morphology. Furthermore, the cells were tightly connected within the central NP area, AF lamella was highly organized, and clear demarcation between NP and AF were maintained at all examined timepoints (Figure 3A). Conversely, progressive degenerative changes were observed in the *Runx1* overexpressing mice over time. At 5M, NP cells appeared scattered peripherally in mice overexpressing *Runx1* (Figure 3C). By 7M, the number of peripherally scattered NP cells increased, and eventually by 10M, NP cells were completely dispersed, accompanied by increased cell clusters and a fibrotic extracellular matrix (Figure 3C). Additionally, the AF tissue progressively exhibited disrupted structure over time, contributing to an irregular NP/AF boundary (Figure 3C). Quantification of histopathological scores confirmed a consistent increase in degeneration scores across the NP (Fold change = 12.0), AF (Fold change = 2.08), and NP/AF boundary (Fold change = 5.0) in *Runx1* overexpression compared to control groups. In addition, the gene expression of *Runx1* was increased in Tam X3 compared to control groups at 10M, confirming a higher level of *Runx1* overexpression (Supplementary Figure 3). Taken together, these findings suggest that *Runx1* overexpression accelerated IVD degeneration in a dose dependent manner, leading to significant structural remodeling and impaired tissue integrity.

### *Runx1* overexpression caused the loss of notochordal cell phenotype

**Figure 4:**
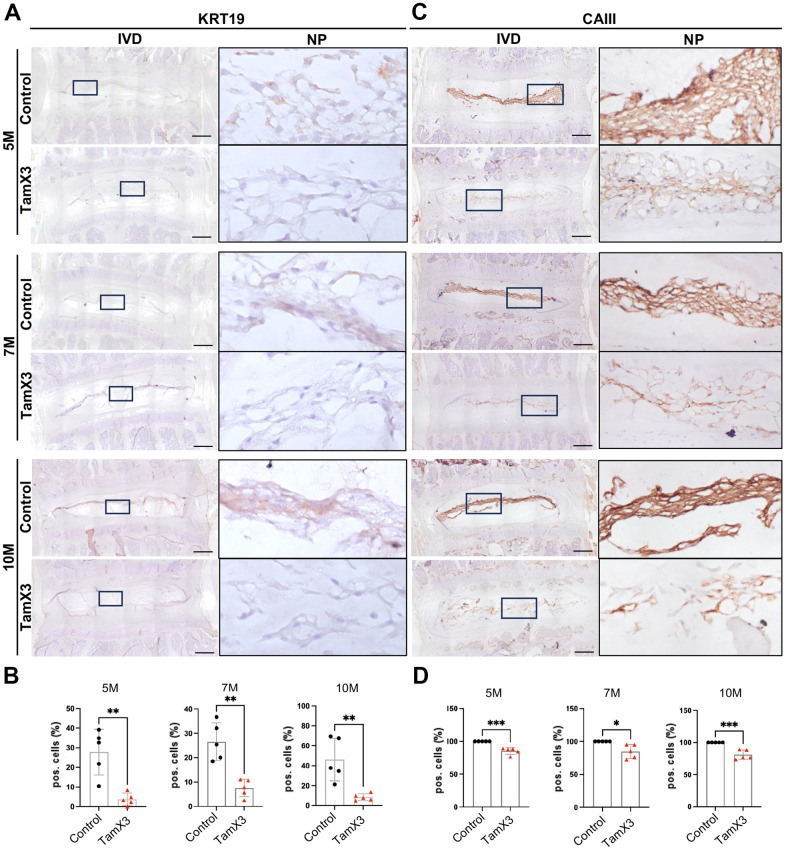
**Runx1 overexpression caused the loss of notochordal cell phenotype.** (**A**, **C**) Representative images of immunohistochemistry staining of notochordal cell marker KRT19 (**A**) and CAIII (**C**) at 5M, 7M, and 10M. Scale bar = 200 μm. Magnified NP areas are shown. Hematoxylin was used as nuclei staining and immunopositivity was labeled brown. (**B**, **D**) Quantification results showing that the KRT19 (**B**) and CAIII (**D**) positive cells were significantly decreased in the NP of Runx1 overexpression mice in all the timepoints examined. n = 5 mice /group. Student’s t-test was performed (* p < 0.05, ** p < 0.01, *** p < 0.001).

The presence of cell clusters and fibrotic ECM in the NP of *Runx1* overexpression mice prompted us to investigate the phenotypic changes of NP cells. To assess this, we examined the expression of two notochordal cell markers cytokeratin 19 (KRT19) and carbonic anhydrase 3 (CAIII) across both genotypes (Figure 4). IHC analysis revealed a significant decrease in the expression of both KRT19 (Figure 4A, 4B; Fold change: 5M = 0.13, 7M = 0.75, 10M = 0.18) and CAIII (Figure 4C, 4D; Fold change: 5M = 0.85, 7M = 0.85, 10M = 0.81) in the NP of *Runx1* overexpression mice, compared to controls at all examined timepoints. These findings indicate that *Runx1* overexpression led to the loss of notochordal cell phenotype, contributing to the progression of IVD degeneration.

### *Runx1* overexpression induced aggrecan degradation in the NP

**Figure 5:**
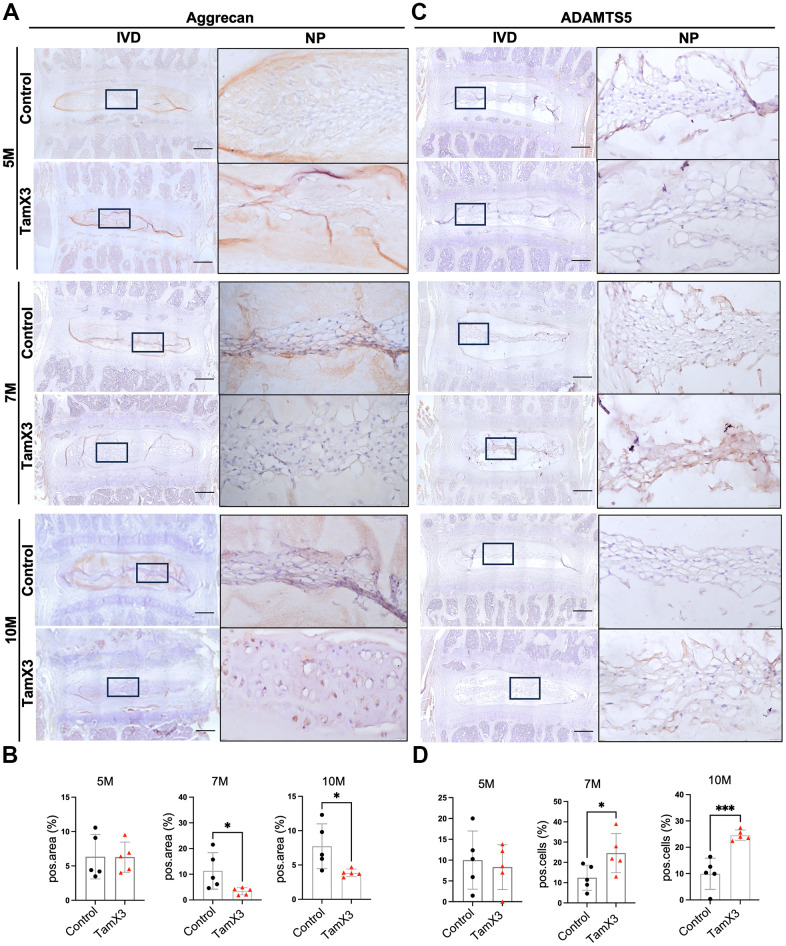
**Runx1 overexpression reduced the accumulation of aggrecan in the NP.** (**A**, **C**) Representative images of immunohistochemistry staining of aggrecan (**A**) and ADAMTS5 (**C**) at 5M, 7M, and 10M. Scale bar = 200 μm. Magnified NP areas are shown. Hematoxylin was used as nuclei staining and immunopositivity was labeled brown. (**B**, **D**) Quantification results showing that the expression of aggrecan (**B**) was significantly decreased at 7M and 10M while the expression of ADAMTS (**C**) was increased in the NP of Runx1 overexpression mice. n = 5 mice /group. Student’s t-test was performed (* p < 0.05, *** p < 0.001).

Aggrecan is the major extracellular matrix molecule in the NP and plays a critical role in maintaining the mechanical properties of the IVD [38]. To determine whether *Runx1* overexpression affected aggrecan deposition, we performed IHC for aggrecan and its degradation enzyme ADAMTS5. In the control NP, aggrecan was abundantly expressed at all timepoints examined. At 5M, no significant changes in the expression of aggrecan were observed between *Runx1* overexpression and control groups (Figure 5A, 5B). However, aggrecan expression was significantly decreased in the NP of *Runx1* overexpression mice at 7M and 10M, compared to the controls (Figure 5A, 5B; Fold change: 7M = 0.3, 10M = 0.53). In contrast to the expression pattern of aggrecan, ADAMTS5 expression levels remained unchanged at 5M but were significantly increased at 7M and 10M in the *Runx1* overexpression group (Figure 5C, 5D; Fold change: 7M = 1.97, 10M = 2.48). Taken together, these results suggest that *Runx1* overexpression disrupted ECM homeostasis by reducing aggrecan deposition and enhancing its degradation through an upregulation of ADAMTS5, contributing to the IVD degeneration.

### *Runx1* overexpression induced collagen remodeling in the NP

**Figure 6:**
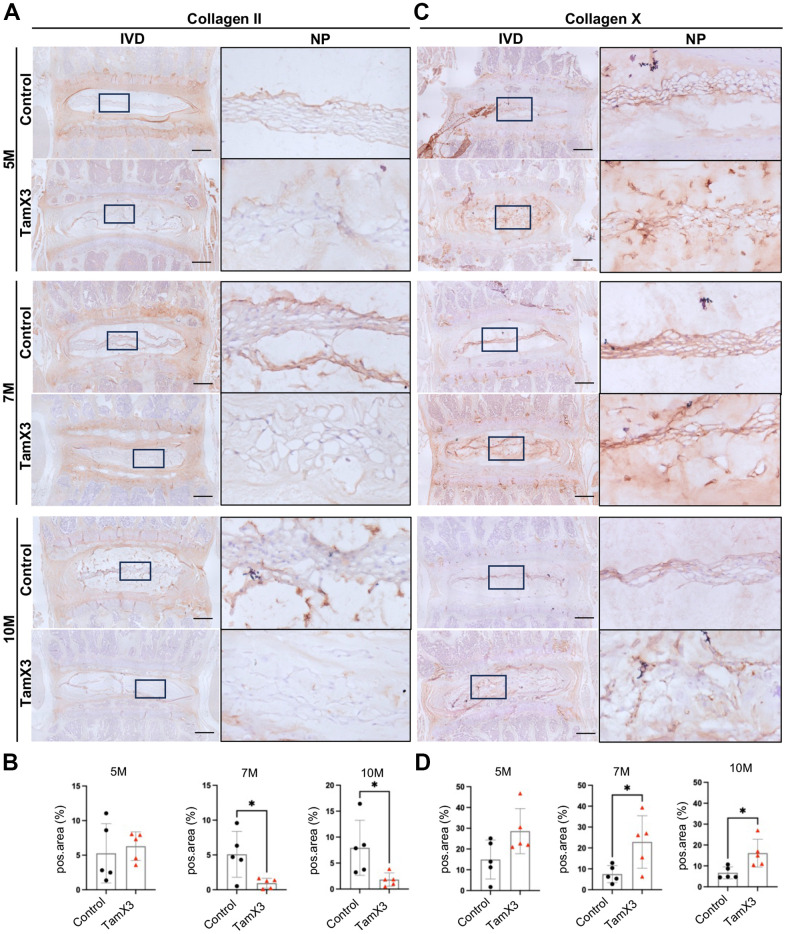
**Runx1 overexpression altered collagen composition in the NP.** (**A**, **C**) Representative images of immunohistochemistry staining of collagen II (**A**) and collagen X (**C**) at 5M, 7M, and 10M. Scale bar = 200 μm. Magnified NP areas are shown. Hematoxylin was used as nuclei staining and immunopositivity was labeled brown. (**B**, **D**) Quantification results showing that the expression of collagen II (**B**) was significantly decreased at 7M and 10M while the expression of collagen X (**C**) was increased in the NP of Runx1 overexpression mice. n = 5 mice /group. Student’s t-test was performed (* p < 0.05).

In addition to aggrecan, collagen II is another major component of ECM in the NP, essential for maintaining the mechanical integrity of the IVD. As IVD degenerates, alterations in collagen composition contribute to impaired mechanical function. Notably, collagen X is often observed in hypertrophic NP cells within degenerated IVDs and is a hallmark of chondrocyte hypertrophy and terminal maturation. Similar to aggrecan, IHC examination for collagen II revealed no significant difference in expression remaining between two control and *Runx1* overexpression groups at 5M (Figure 6A, 6B). However, its expression was significantly decreased in the NP of *Runx1* overexpression mice at 7M and 10M, compared to the controls (Figure 6A, 6B; Fold change: 7M = 0.23, 10M = 0.18). In contrast, collagen X remained unchanged at 5M but was markedly increased at 7M and 10M in the *Runx1* overexpression group (Figure 6C, 6D; Fold change: 7M = 3.06, 10M = 2.41). Moreover, the gene expression of *Col10a1* and *Vegfa* was increased in *Runx1* overexpression group (Supplementary Figure 4), further indicating a shift towards a hypertrophic phenotype. Collectively, these results suggested that *Runx1* overexpression caused collagen remodeling, which may accelerate IVD degeneration and impair disc function.

### *Runx1* overexpression promoted NP cell senescence

**Figure 7:**
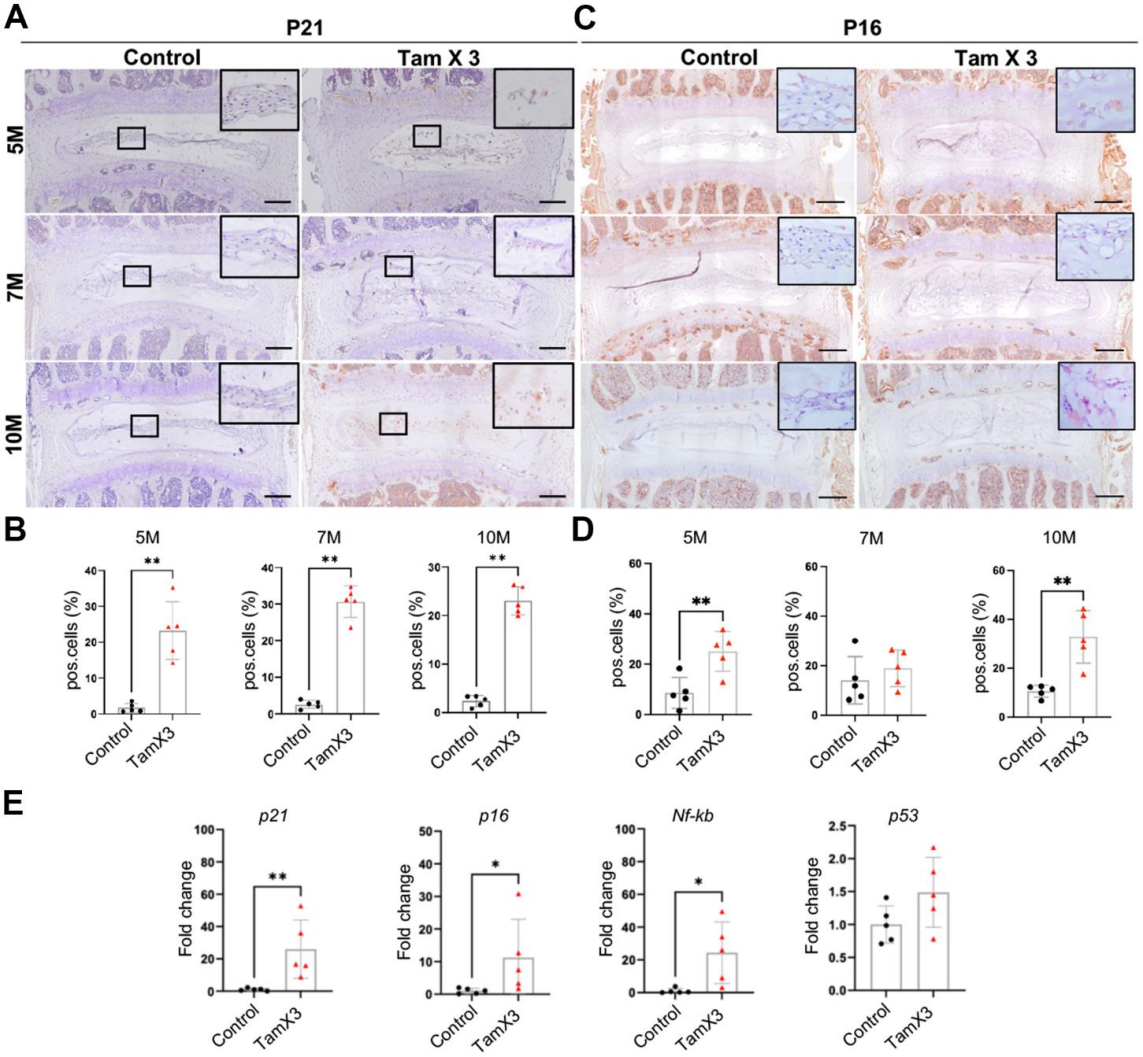
**Runx1 overexpression induced NP cell senescence.** (**A**, **C**) Immunohistochemistry staining for P21 (**A**) and P16 (**B**) was conducted to detect the presence of cell senescence in the NP. Scale bar = 200μm. (**B**, **D**) Quantification analysis showed that substantial increase in the ratio of P21 positive cells in Runx1 overexpression mice. P16 positive cells were increased significantly at 5M and 10M but not at 7M. n = 5 mice /group. Student’s t-test was performed (** p < 0.01). (**E**) The gene expression analysis demonstrated the increased levels of p21, p16, and Nf-kb in Runx1 overexpression mice at 10M. The expression level of p53 remained unchanged in the Runx1 overexpression mice. n = 5 mice /group. Student’s t-test was performed (** p < 0.01, * p < 0.05).

The observed morphological and phenotypic changes in response to *Runx1* overexpression compelled us to explore the mechanisms underlying the induction and acceleration of IDD in aging mice. Given that senescence is a key contributor to IVD aging and degeneration [39], we conducted IHC to examine the expression of cell senescence markers P21 and P16. The IHC analyses revealed a substantial increase in the number of P21-positive cells in the NP of *Runx1* overexpression mice (Figure 7A, 7B, Fold change: 5M = 13.53, 7M = 11.76, 10M = 9.82) at all timepoints examined. Similarly, an increase in the percentage of P16-positive cells was observed in the NP of *Runx1* overexpressing mice at 5M and 10M, but not at 7M of age, suggesting that a complex, stage-dependent regulation of senescence in NP cells (Figure 7C, 7D, Fold change: 5M =2.92, 10M =3.10). To further validate these findings, qPCR analysis of RNA extracted from NP tissues harvested by laser capture microdissection (LCM) confirmed a significant upregulation of transcript levels of the senescence markers p21 (fold change: 26.0), *p16* (fold change: 11.2)*,* and *Nf-kb* (fold change: 24.35) in *Runx1* overexpression mice compared to controls (Figure 7E). However, the mRNA levels of p53, a key regulator of both senescence and apoptosis, were not significantly changed between the two genotypes. The lack of p53 activation led us to evaluate whether *Runx1* overexpression impacted cell apoptosis. TUNEL assay demonstrated no significant difference in the percentage of apoptotic cells in the NP between *Runx1* overexpression and control mice at all timepoints examined (Supplementary Figure 5). Taken together, *Runx1* overexpression promoted premature and sustained cellular senescence in NP cells, contributing to the progression of IVD degeneration.

## DISCUSSION

The mechanisms underlying aging-related disc degeneration remain elusive. In this study, we used a genetic mouse model to define the role of the Runx1 transcription factor in this process. The rationale for examining the role of Runx1 in degenerative discs stems from our previous transcriptomic analyses of human degenerated NP cells in which we identified Runx1 as a putative regulator of human IVD degeneration [36]. Moreover, genetic evidence linking Runx1 to disc degeneration was reported by Sato and colleagues, showing that overexpression of Runx1 in Collagen II positive cells led to severe developmental abnormalities in the IVD [35]. To establish a functional role of Runx1 in aging discs, we employed a Tamoxifen-inducible transgenic mouse approach to over-express *Runx1* specifically in NP cells. Tamoxifen was administered at 4 weeks of age, when the NP is already established, to avoid potential interference with early spinal development. The effects of *Runx1* overexpression on IVD degeneration were evaluated at 5M, 7M, and 10M. Our findings demonstrated that NP specific *Runx1* overexpression induced early onset and progressive IVD degeneration by promoting a phenotypic shift from notochordal cells to hypertrophic chondrocyte-like cells, ECM remodeling, and a prosenescent phenotype. This study highlights a novel role of Runx1 in regulating disc health and aging.

The optimal tamoxifen dose required to efficiently induce Krt19-CreERT-mediated recombination in NP cells using the Rosa26 mT/mG mice was previous established [40]. In the study by Mohanty and colleagues, a single dose of tamoxifen resulted in only 4.8% of NP cells expressing mGFP in the lumbar IVD. However, the recombination efficiency significantly increased to 77.2% after two doses and reached 92.0% after three doses of tamoxifen [40]. Given the wide range of response to different tamoxifen injections, we elected to test tamoxifen dosing modalities in our transgenic mouse and examine their effects on Runx1 overexpression and its consequences. Our study revealed a dose-dependent increase in *Runx1* overexpression in *Krt19*^CreERT^; *Rosa26*-*Runx1* mice, with higher level of *Runx1* expression in response to increasing tamoxifen injections. Notably, mice receiving three times of tamoxifen injections exhibited severe IVD degeneration compared to those receiving two injections, suggesting a dose-dependent relationship between *Runx1* expression and the severity of IVD degeneration.

The loss of notochordal cells and their transition to chondrocyte-like mature NP is a key event linked to the initiation and progression of IVD degeneration in humans [41, 42]. This phenotypic shift leads to ECM remodeling, reducing the tissue structure integrity and impairing IVD function. In contrast, mice typically maintain the notochordal NP cell population throughout most of their lifespan, making them less susceptible to IVD degeneration compared to humans [43]. One of our observations here was that mice postnatally overexpressing *Runx1* in the NP displayed a pronounced decrease in notochordal cell markers as early as 5M. This was accompanied by the presence of peripherally scattered, round-shaped NP cells. Such phenotype was reported in the lumbar discs of wild type mice at a much more advanced age (14M) [7]. Our hypothesis that *Runx1* overexpression may accelerate disc aging was further reinforced by our 10M data revealing dispersed NP cells with a fibrotic ECM characterized by reduced aggrecan and COL II levels, alongside increased COL X expression in our transgenic compared to control mice. A previous study reported that a reversal in the COL II to COL X ratio strongly correlates to IVD degeneration [44]. This ECM remodeling, driven by phenotypic alterations in NP cells, suggests that *Runx1* overexpression accelerated the progression of age-related IVD degeneration and may play a role in promoting the differentiation of notochordal cells into chondrocyte-like cells during IDD. Lin and colleagues previously reported that the injection of Runx1 mRNA without carrier resulted in opposite structural changes compared to the injection of nanomicelle-encapsulated Runx1 mRNA into degenerated IVDs using a rat puncture IDD model [45]. While both injection methods increased disc height index compared to the puncture only group, delivery of Runx1 mRNA without nanomicelles exacerbated inflammation and macrophage infiltration in the punctured IVDs. Our interpretation of these data is that uncontrolled Runx1 expression may contribute to an inflammatory response in an acute injury model. However, it remains unclear whether the observed reduction in inflammation was due to the controlled release of Runx1 mRNA or nanomicelles themselves, as they did not provide an empty nanomicelle control group. In contrast, our genetic model allows control over both expression levels and tissue targeting at the cellular level. To investigate potential dose-dependent effects of Runx1 on IDD, we took advantage of our inducible system by varying the dose of tamoxifen, thereby modulating Runx1 expression. The other possible explanation of their outcomes observed by Lin et al, would be a potential temporal regulation at a specific dosage of Runx1 that elicited the observed phenotype in their surgical model. Perhaps, further mechanistic studies in their models would help to validate these outcomes, which our genetic model has functionally addressed.

*Runx1* is known to regulate cell fate and differentiation by modulating gene expression, interacting with other transcription factors, and influencing epigenetic states across various cell types, including neurons [46] and myeloid cells [47]. Work from our own laboratory has further established *Runx1* as a regulator of chondrogenic and osteoclastogenic differentiation, both *in vitro* and *in vivo* [31, 33, 48]. Runx1 has been recognized as having pleiotropic effects beyond its initially characterized role in hematopoiesis [49]. In this study, we present evidence that the expression of *Runx1* in disc tissue may be functioning temporally to control aging-associated disc degeneration.

Our study also implicates *Runx1* as a potential inducer of NP cell senescence rather than cell apoptosis, suggesting a likely mechanism by which Runx1 accelerates ECM degradation and promotes NP tissue aging. Previous studies have shown that *Runx1* can induce a senescence-like growth arrest in primary mouse embryonic fibroblasts (MEFs) by slowing cell cycle progression and reducing proliferation [50, 51]. It is well established that accumulation of senescent cells in the IVD is strongly associated with disc aging and degeneration. Indeed, studies have shown that NP samples from older IDD patients exhibited a higher expression of P21 compared to younger individuals [52]. The non-dividing and metabolically active senescent cells secrete a variety of pro-inflammatory cytokines and matrix degrading enzymes, contributing to the breakdown and degeneration of the disc tissue. Consistent with these findings, our study revealed that *Runx1* overexpression in the NP significantly upregulated key senescence markers, including *p21*, *p16,* and *Nf-kb*. Furthermore, we observed increased expression of ADAMTS5 and decreased aggrecan in the NP of *Runx1* overexpression mice. These findings further support that *Runx1* overexpression can drive a pro-senescent and catabolic microenvironment, thereby exacerbating ECM degradation and contributing to tissue aging.

One noteworthy limitation of our study is its primary focus on structural and molecular changes without considering potential alterations in the mechanical properties of the disc over time as well as any potential pain-related behavior. These are important outcomes that warrant further investigation. We anticipate that future studies incorporating comprehensive functional assessments, including biomechanical testing and behavioral analyses, will provide a complete understanding of the function of *Runx1* in disc health and degeneration.

In summary, our findings highlight the important role of *Runx1* in regulating disc health and reveal this pleiotropic transcription factor as a potential therapeutic target for treating IVD degeneration and aging. We speculate that despite these encouraging results, future studies with wider experimental groups and increased outcome measures as well as investigating long-term effects of *Runx1* overexpression in IVD aging and degeneration will be required to fully elucidate its role in chronic disc pathology and perhaps generalize this novel concept that Runx1 may control disc aging through regulation of NP cell senescence.

## MATERIALS AND METHODS

### Generation of *Krt19*^CreERT^; *Rosa26*-*Runx1* mice

All procedures in mice were conducted under the approved IACUC protocol # V010-23. Homozygous *Krt19*^CreERT^ mice (Strain #:026925) were obtained from The Jackson Laboratory. *Rosa26*-*Runx1* transgenic mice were generated as previously described [30]. Briefly*, Rosa26-Runx1* mice contain a stop cassette flanked by LoxP sites and downstream the *Runx1* sequence which were inserted into the native *Rosa26* gene locus in the C57BL/6J mice.

To produce inducible *Runx1* overexpressing mice, homozygous *Krt19*^CreERT^ mice were crossed with *Rosa26*-*Runx1* transgenic mice and obtained *Krt19*^CreERT^; *Rosa26*-*Runx1* mice. Genotyping using tail DNA was performed to identify mice carrying both transgenes. In this study, *Rosa26*-*Runx1* transgenic mice were used as control, and all experiments were performed with male mice.

### Tamoxifen induction of *Runx1* overexpression in the NP

Tamoxifen was employed to induce Cre recombinase activity in *Krt19*^CreERT^; *Rosa26*-*Runx1* mice, and *Rosa26*-*Runx1* transgenic mice as control. The dose and administration protocol were adapted from a previously established procedure [53]. Tamoxifen (Sigma Aldrich, T2859) was dissolved in Corn oil (Sigma Aldrich, C8267) at a concentration of 20 mg/ml. The solution was protected from light and incubated in a water bath set at 55° C overnight to ensure complete dissolution. Intraperitoneal (IP) injections in the mice were performed using a 26G needle to mice at 4 weeks old. The injection dosage was set at 2 mg/10 g body weight. In the two-injection group, tamoxifen was administered once daily for two consecutive days. In the three-injection group, tamoxifen was administered once daily for three consecutive days.

### NP tissue harvesting

Mouse NP tissues were harvested following necropsy from both control and *Krt19*^CreERT^; *Rosa26*-*Runx1* mice, treated with tamoxifen through IP injections as described above. Following necropsy, the spinal columns including the coccyx were dissected out, and L4-5 were used for subsequent histological analysis, while NP tissues from the remaining spine were meticulously dissected under a microscope and promptly transferred to a pre-chilled homogenization tube for subsequent molecular analyses.

### RNA isolation

To ensure the extraction of high-quality RNA from the dissected NP tissue, TRIzol Reagent (Invitrogen, 15596026) was added to the homogenization tube, and RNA isolated following the manufacturer’s instructions. RNA quality and concentration were assessed using the NanoDrop One instrument (Invitrogen). Samples were stored in -20° C for future gene expression evaluation.

### Quantitative PCR (qPCR) analysis

For gene expression analysis, qPCR was conducted. Complementary DNA (cDNA) synthesis was performed using the High-Capacity cDNA Reverse Transcription Kit (Applied Biosystems, 4368814). A total of 1μg of isolated RNA was converted into cDNA. Subsequently, qPCR was carried out using the PowerUp SYBR Green Master Mix (Applied Biosystems, A25742). Gene expression levels were normalized to beta-actin, serving as an internal control, with delta-delta-Ct method. The primer sequences employed in the qPCR analysis are provided in Table 1.

**Table 1:** qPCR primer sequence.

**Gene**	**Forward**	**Reverse**
*Actb*	5’- CTAAGGCCAACCGTGAAAAG -3’	5’- AGCCTGGATGGCTACGTACA -3’
*Runx1*	5’- ACTGGCGCTGCAACAAGA -3’	5’- CATCGTTGCCTGCCATGAC -3’

### Western blot analysis

For protein isolation, NP tissues were transferred to a tube, and T-PER™ Tissue Protein Extraction Reagent (Thermo Fisher Scientific, 78510) was added. The protein was extracted following our previously described method [54]. Equal amounts of protein (20μg) were loaded onto a Mini-PROTEAN TGX Stain-Free Protein Gel (BIORAD, 4568024), subjected to electrophoresis, and transferred to a PVDF membrane. The blot was then incubated in 5% milk for one hour, followed by the anti-mouse RUNX1 primary antibody incubation (1:500, Abcam, ab229482) overnight at 4° C. After washing, the membrane underwent probing with a secondary anti-rabbit IgG, HRP-linked antibody (Cell Signaling, 7074), and protein bands were visualized using an ECL Select Western Blotting Detection Reagent (Cytiva, GERPN2235). The bands were detected with the ChemiDoc MP Imaging System (BIORAD).

### Radiological assessment of IDD in mouse

Intervertebral disc height was evaluated by measuring the disc height index (DHI) at 5M, 7M, and 10M, following the method described previously [37].

Control and *Krt19*^CreERT^; *Rosa26*-*Runx1* mice were anesthetized, and X-rays were obtained using a XPERT 40 System (KUBTEC). The assessment was specifically conducted at the L4-5 level. Disc height index was analyzed by measuring the disc height and corresponding vertebral heights on lateral lumbar spine X-rays (details provided in Figure 2). The measurements of the data were blinded to both the age and genotype of mice.

### Histopathological assessments of IVD sections

Histopathological assessment of IVD sections was performed on control and *Krt19*^CreERT^; *Rosa26*-*Runx1* mice at 5M, 7M, and 10M. Lumbar spines were harvested and fixed in 10% Neutral Buffered Formalin (Fisher Scientific, 427098) for 24 hours. Paraffin-embedded sections (5μm) were obtained, and Hematoxylin and Eosin (H&E) staining was utilized to evaluate general tissue morphology. Bright-field microscopy (BX63, OLYMPUS) was used for imaging. All samples were evaluated at the L4-5 level. The degree of degeneration was assessed using histological scores measured according to the previously published system [55] and one midcoronal section per group was used for grading. The summary of histopathological scores for each sample was provided in Supplementary Table 1.

### Immunohistochemistry (IHC)

Histological sections were deparaffinized with xylene and hydrated with a series of graded ethanol/H_2_O solutions and antigen retrieval performed using hyaluronidase, citrate buffer, or proteinase kinase. For detecting the expression of aggrecan, ADAMTS5, type II collagen, and type X collagen, the hyaluronidase method was specifically used. Briefly, the sections were incubated with 0.8% hyaluronidase at 37° C for 1 hour. For the citrate buffer method (used for detecting the expression of cytokeratin 19, CAIII, and P16), the sections were submerged in the buffer and heated in microwave until the buffer boiled. The sub-boiling temperature was maintained for 20 minutes. To detect P21 proteins, the proteinase kinase method was used. Briefly, the sections were incubated with 20μg/ml proteinase kinase in a 37° C humidified chamber for 30 minutes. Subsequently, sections were treated with a 3% hydrogen peroxide solution for 20 minutes to block endogenous peroxidase activity. Non-specific binding was blocked by incubating sections in 10% goat serum (Abcam, ab7481) for 1 hour at room temperature. Primary antibodies, KRT19 (dilution ratio 1:200, Cell signaling technology, 12434), CAIII (dilution ratio 1:200, Proteintech, 15197-1-AP), Aggrecan (1:200, Bioss, BS-1223R), ADAMTS5(dilution ratio 1:100, Abcam, ab41037), collagen II (dilution 1:200, ROXKLAND, 600401104), collagen X (dilution ratio 1:200, Abcam, ab58632), P16 (1:200, Thermo Fisher Scientific, PA520379) and P21 (dilution ratio 1:25, Novus Biologicals, NB100-1941), were applied to the section and incubated at 4° C overnight. Next day, SignalStain Boost IHC Detection Reagent (Cell Signaling, Rabbit 8114) was applied, followed by a 30-minute incubation at room temperature. For chromogenic detection, DAB substrate kit (Cell Signaling, 8059) was utilized according to the manufacturer’s instructions, and counterstaining was performed with hematoxylin. For cytokeratin 19, CAIII, P16 and P21 IHC quantification, positive cells and total cells in the NP area were counted to calculate the percentage of positive cells. For aggrecan, ADAMTS5, collagen II, and collagen X, positive area in the NP was quantified.

### TUNEL (Terminal deoxynucleotidyl transferase dUTP nick-end labeling) assay

Apoptotic cells in the IVD were assessed using a commercially available TUNEL assay kit according to the manufacturer’s protocol (Thermo Fisher Scientific, C10618). Briefly, paraffin-embedded sections (5μm) were deparaffinized as described previously. Sections were permeabilized with proteinase K for 15 minutes at room temperature. Positive control samples were treated with DNAse to fragment DNA for 30 minutes. The sections were then incubated with the TUNEL reaction mixture to label fragmented DNA in apoptotic cells in a humidified chamber for 1 hour at 37° C. Following incubation, sections were incubated with Click-iT Plus TUNEL assay with Alexa Fluro 488 to detect the fragmented DNA. The slides were counterstained with DAPI (Abcam, ab104139) and imaged using a fluorescence microscope (BX63, OLYMPUS). Quantification of TUNEL-positive cells was performed in the NP regions using Image J software.

### Laser capture microdissection (LCM)

To isolate RNA specifically from the NP area under direct microscopic visualization, a laser capture microdissection (LCM) system was employed [56]. PEN Frame Slides (Leica Microsystems, 11600289) were decontaminated by sterilizing under UV light for 30 minutes before use. Paraffin-embedded block was sectioned into 15μm sections, mounted on pretreated membrane slides, and incubated at 60° C for two hours. The sections were deparaffinized with xylene (20 seconds for three times) and hydrated using a graded series of ethanol (30 seconds for twice in 100%, 30 seconds for twice in 95%, 30 seconds for twice in 70%), then air-dried until complete water evaporation. The membrane slides were placed on a laser microdissection microscope (Leica Microsystems, DFC7000T), and the NP area at the L4-5 level was selectively laser-cut. Following microdissection, total RNA was purified using the RNeasy FFPE Kit (Qiagen, 73504) following the manufacturer’s instructions. Subsequently, RNA purity and concentration were assessed using a NanoDrop One instrument (Invitrogen).

### Preamplification of RNA and qPCR with LCM samples

A total of 10 ng of isolated RNA via LCM was preamplified with Prelude™ One-Step PreAmp Master Mix (Takara Bio, 638553) following the manufacturer’s instruction. Amplified products were used for downstream qPCR using the PowerUp SYBR Green Master Mix (Applied Biosystems, A25742). Gene expression levels were normalized to glyceraldehyde-3-phosphate dehydrogenase (*Gapdh*), serving as an internal control. The primer sequences used in the qPCR analyses following LCM are listed in Table 2.

**Table 2:** qPCR primer sequence for LCM.

**Gene**	**Forward**	**Reverse**
*Gapdh*	5’- CTCTCTGCTCCTCCCTGTTC -3’	5’- ACACCGACCTTCACCATTTT -3’
*Runx1*	5’- CAACAAGACCCTGCCCATC -3’	5’- TGACCAGAGTGCCATCCG -3’
*Col10a1*	5’- CATCCCATACGCCATAAAGAGT -3’	5’- TCTCCTCTTACTGGAATCCCTTT -3’
*Vegfa*	5’- CGTCAGAGAGCAACATCACC -3’	5’- CCTATGTGCTGGCTTTGGTG -3’
*p53*	5’- TCTCCGAAGACTGGATGACT -3’	5’- AGGCTGATATCCGACTGTGA -3’
*p21*	5’- ATATCCAGACATTCAGAGCCAC -3’	5’-GACATCACCAGGATTGGACAT -3’
*p16*	5’- GAGGATCTTGAGAAGAGGGC -3’	5’- CCATCATCATCACCTGGTCC -3’
*Nf-kb*	5’- CTGACCATGGACGATCTGTT -3’	5’- TGGCTCTGAGGGAAAGATGA -3’

### Statistical analysis

All experimental graphs represent data obtained from five independent mice per group with two technical duplicates as detailed in the figure legends. Data are presented as means ± standard deviation (SD). Statistical significance was evaluated using either Student t-test or a one-way ANOVA followed by Tukey’s post hoc test using GraphPad Prism 9. Statistically significant differences were considered based on a minimum p-value ≤ 0.05.

## Supplementary Material

Supplementary Figures

Supplementary Table 1
