# Supplementary material for: Runx1 overexpression induces early onset of intervertebral disc degeneration
Source: Aging (Albany NY). 2025 Sep 8;17(9):2369–91. doi: 10.18632/aging.206316 (PMC12517211; doi:10.18632/aging.206316)
Supplement: Supplementary Figures [file aging-17-9-206316-s001.pdf]

SUPPLEMENTARY FIGURES

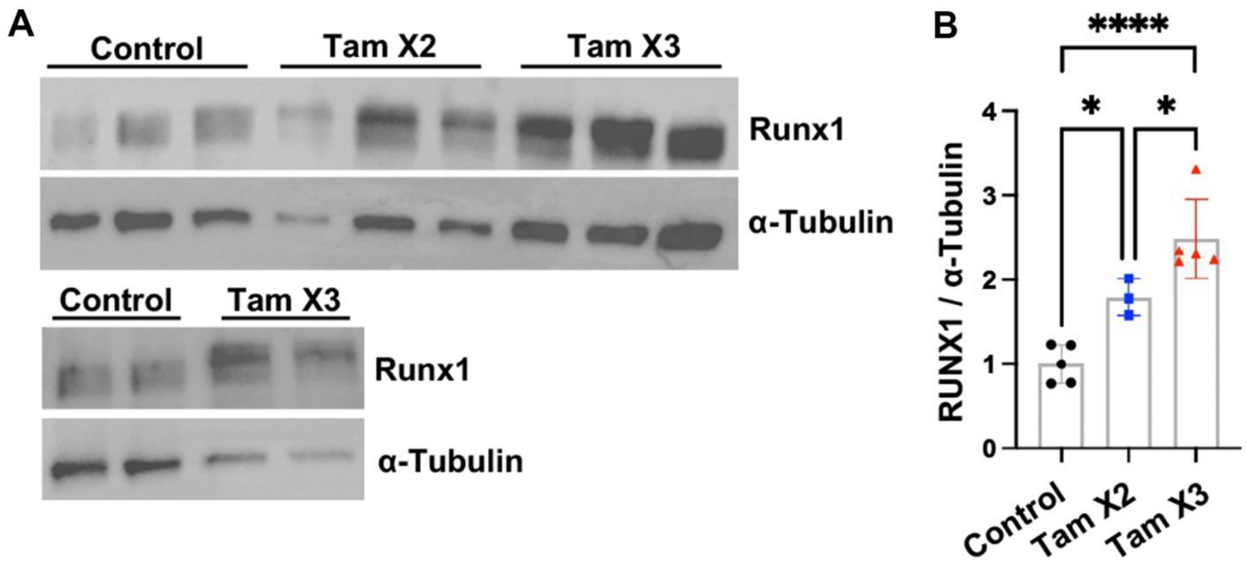

Supplementary Figure 1. The Runx1 protein expression was increased with tamoxifen injections in a dose-dependent manner. (**A**) Western blot analysis was performed on the NP of control, Tam X2, and Tam X3 groups. (**B**) Quantification analysis showed the increased expression of RUNX1 in the NP tissue from the Tam X3 group, compared to Tam X2 and control groups. Control: n = 5 mice. Tam X2: n = 3 mice. Tam X3: n = 5 mice. One-way ANOVA followed by Tukey's post-test was performed. (**** p < 0.0001, * p < 0.05).

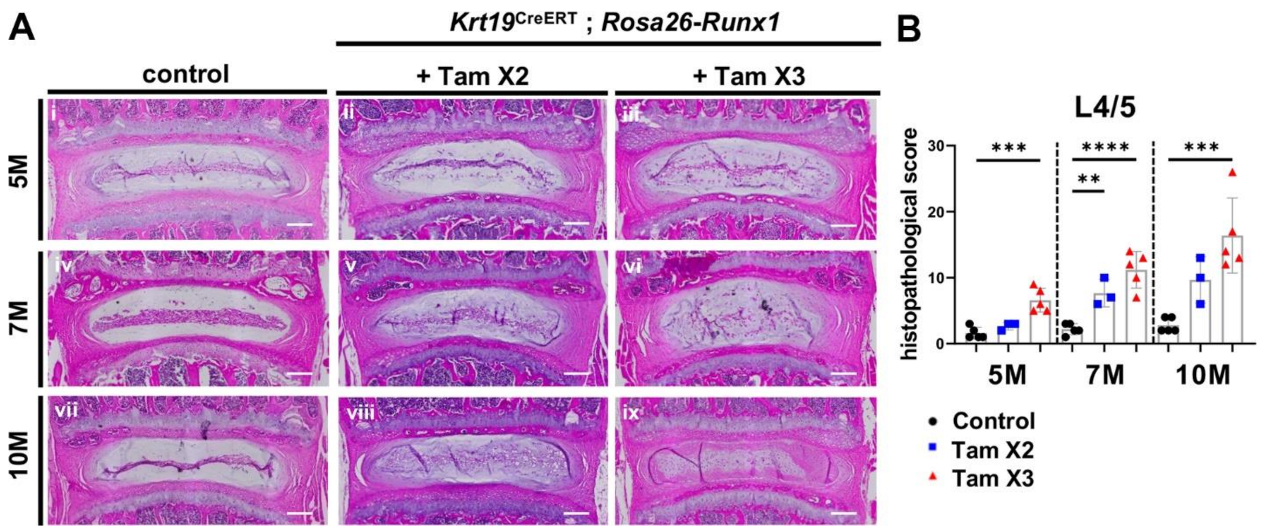

Supplementary Figure 2. Runx1 overexpression induced age-related intervertebral disc degeneration. (**A**) Representative images of H&E staining showing the severity of IVD degeneration is dose-dependent of Runx1 expression, with severe degenerative changes in the Tam X3 compared to Tam X2 and control groups at 5M, 7M, and 10M. Scale bar = 200 µm. Note that the representative images displayed for the control and Tam X3 groups are the same as those shown in Figure 3A. (**B**) Quantification analysis showed a dose dependent increase in histological scores in the IVDs with Runx1 overexpression. Control and Tam X3: n = 5 mice/group. Tam X2: n = 3 mice/group. One-way ANOVA followed by Tukey's post-test was performed. **** p < 0.0001, *** p < 0.001, ** p < 0.01.

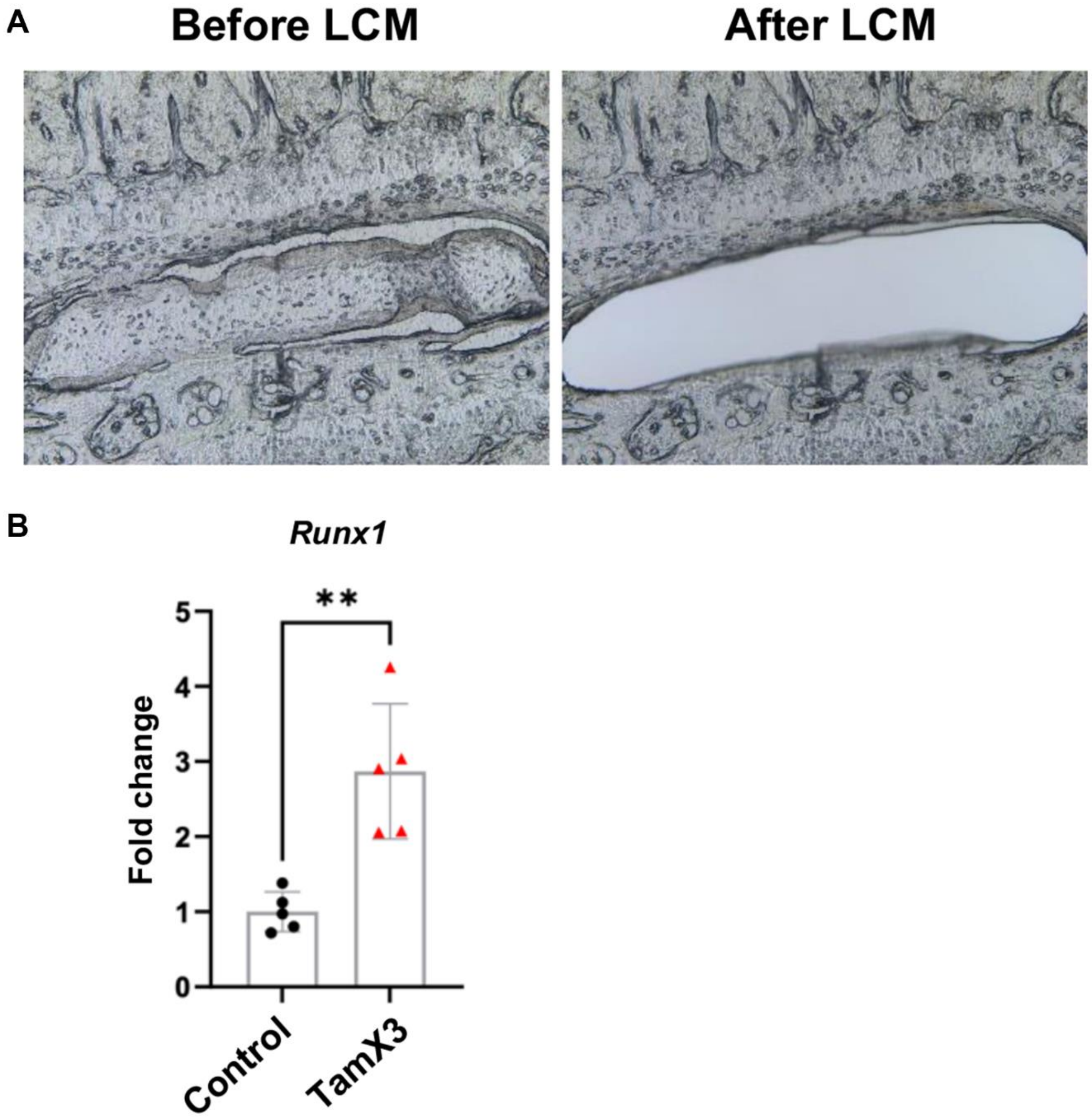

Supplementary Figure 3. qPCR analyses using laser capture microscope (LCM). (**A**) Representative images showing the NP region collected using LCM. (**B**) The expression level of Runx1 remained elevated at 10M in the Krt19CreERT; Rosa26-Runx1 mice after tamoxifen induction at 4 weeks of age. n = 5 mice /group. Student t-test was performed. (** $p < 0.01$).

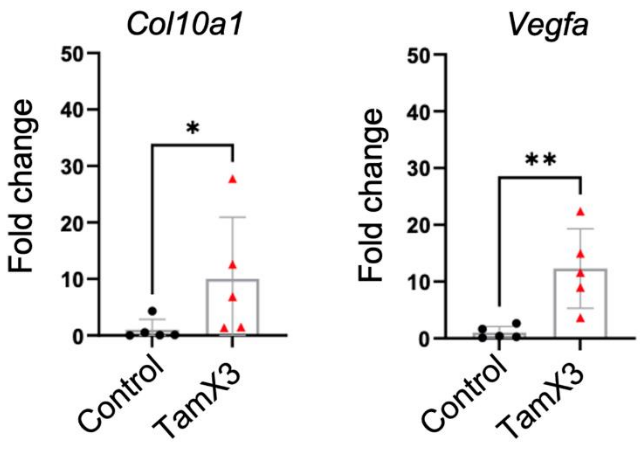

Supplementary Figure 4. Runx1 overexpression induced an increase in the gene expression of Col10a1 and Vegfa. Gene expression analysis on NP tissues using laser capture microscope confirmed that the expression level of the hypertrophic markers collagen type X alpha 1 chain (Col10a1) and vascular endothelial growth factor A (Vegfa) was upregulated in Runx1 overexpression mice. n = 5 mice /group. Student's t-test was performed (* $p < 0.05$, ** $p < 0.01$).

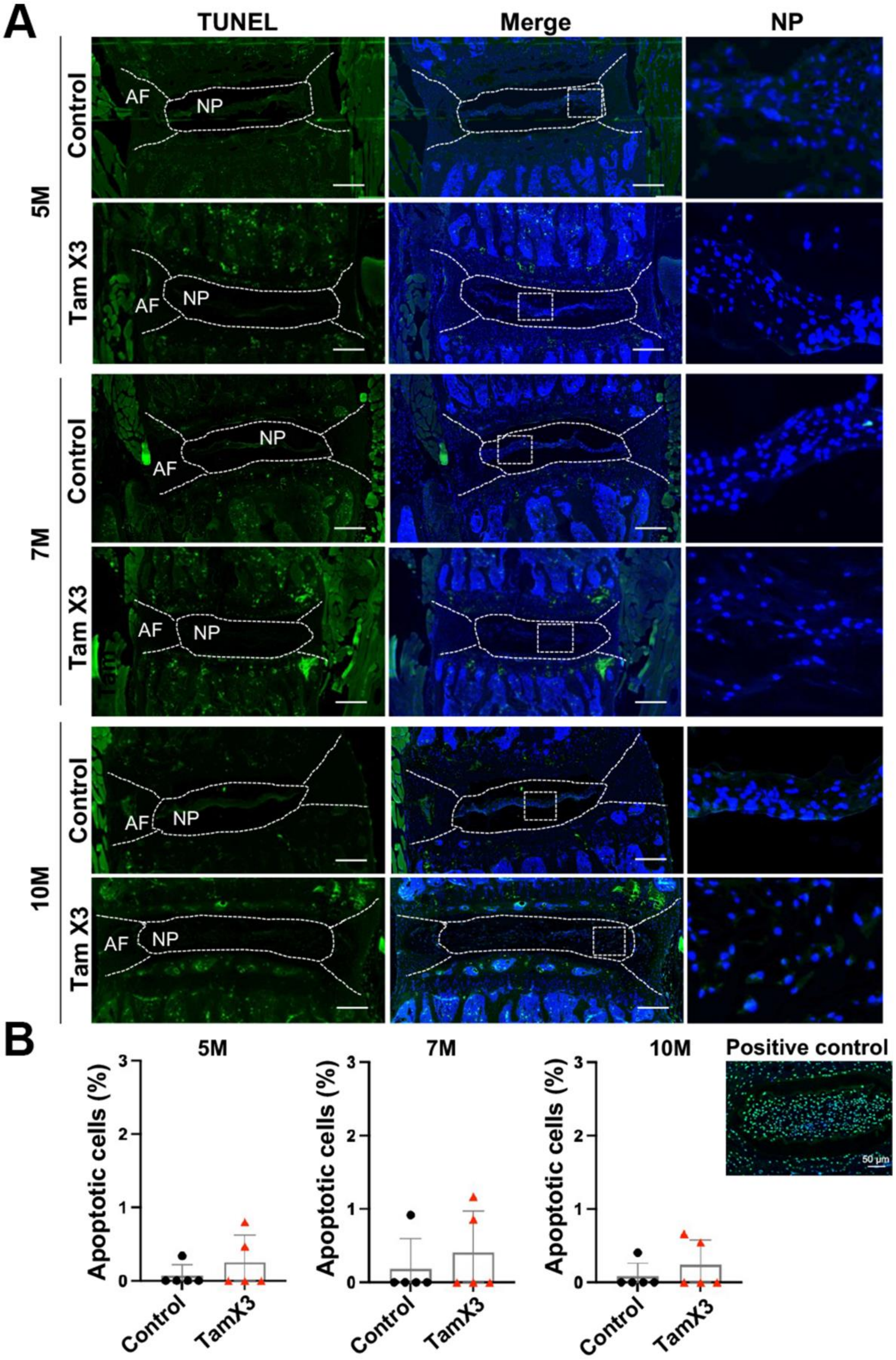

Supplementary Figure 5. Runx1 overexpression did not affect NP cell apoptosis. (**A**) Representative images of TUNEL assay on control and Runx1 overexpression mice at 5M, 7M, and 10M. Scale bar = 200 μm. (**B**) Quantification of TUNEL assay showing that no significant changes were detected between two genotypes at all timepoints examined. n=5/group. Summary of histopathological score of control and Tam mice stained with H&E in L4-L5
